# Supplementary material for: Conditional deletion of Nedd4-2 in lung epithelial cells causes progressive pulmonary fibrosis in adult mice
Source: Nat Commun. 2020 Apr 24;11:2012. doi: 10.1038/s41467-020-15743-6 (PMC7181726; doi:10.1038/s41467-020-15743-6)
Supplement: Supplementary file 1 — Supplementary Information [file 41467_2020_15743_MOESM1_ESM.pdf]

Supplementary information

Conditional deletion of *Nedd4-2* in lung epithelial cells causes progressive pulmonary fibrosis in adult mice

Duerr et al.

Supplementary Figures

Supplementary Figure 1

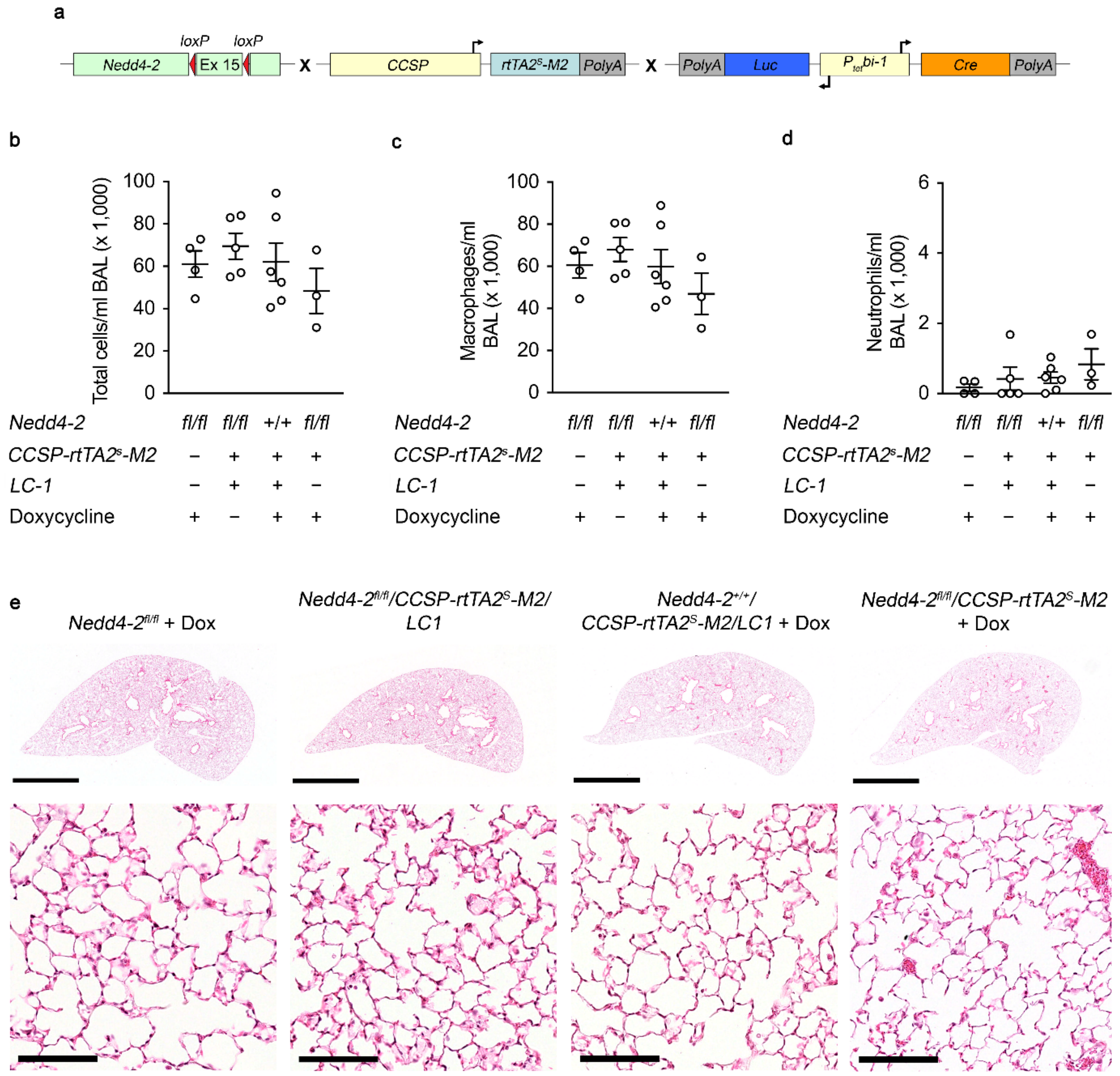

Supplementary Figure 1. Constructs used for conditional deletion of Nedd4-2 in lung epithelial cells and assessment of pulmonary phenotypes of mice used as controls. (**a**) Schematic representation of the transgenic cDNA constructs and the floxed genomic locus necessary for conditional deletion of *Nedd4-2* in lung epithelial cells of triple-mutant *Nedd4-2*$^{fl/fl}$*/CCSPrtTA2*^{S}*-M2/LC1* mice. (**b**-**e**) Total cell counts (**b**), number of macrophages (**c**) and neutrophils (**d**) in BAL and representative morphology of hematoxylin and eosin stained lung sections (upper row, scale bars, 2.5 mm; lower row, scale bars, 100 µm) (**e**) were assessed in mice with the different genotypes that served as controls to exclude potential off-target effects of the expression system: i) *Nedd4-2*$^{fl/fl}$ mice induced with doxycycline for 4 months served as control for side effects of long-term doxycycline

treatment (n = 4); ii) age-matched *Nedd4-2$^{fl/fl}$/CCSP-rtTA2^{S}-M2/LC1* mice without doxycycline-induction served as control for leakiness of rtTA-mediated gene expression (n = 5), iii) doxycycline-induced (4 months) *CCSP-rtTA2^{S}-M2/LC-1* mice served as control for side effects of long-term Cre recombinase expression (n = 6); and iv) doxycycline-induced (4 months) *Nedd4-2$^{fl/fl}$/CCSP-rtTA2^{S}-M2* mice served as control for long-term expression of the rtTA. (n = 3). Data are shown as mean ± S.E.M.. Source data are provided in the Source Data file.

Supplementary Figure 2

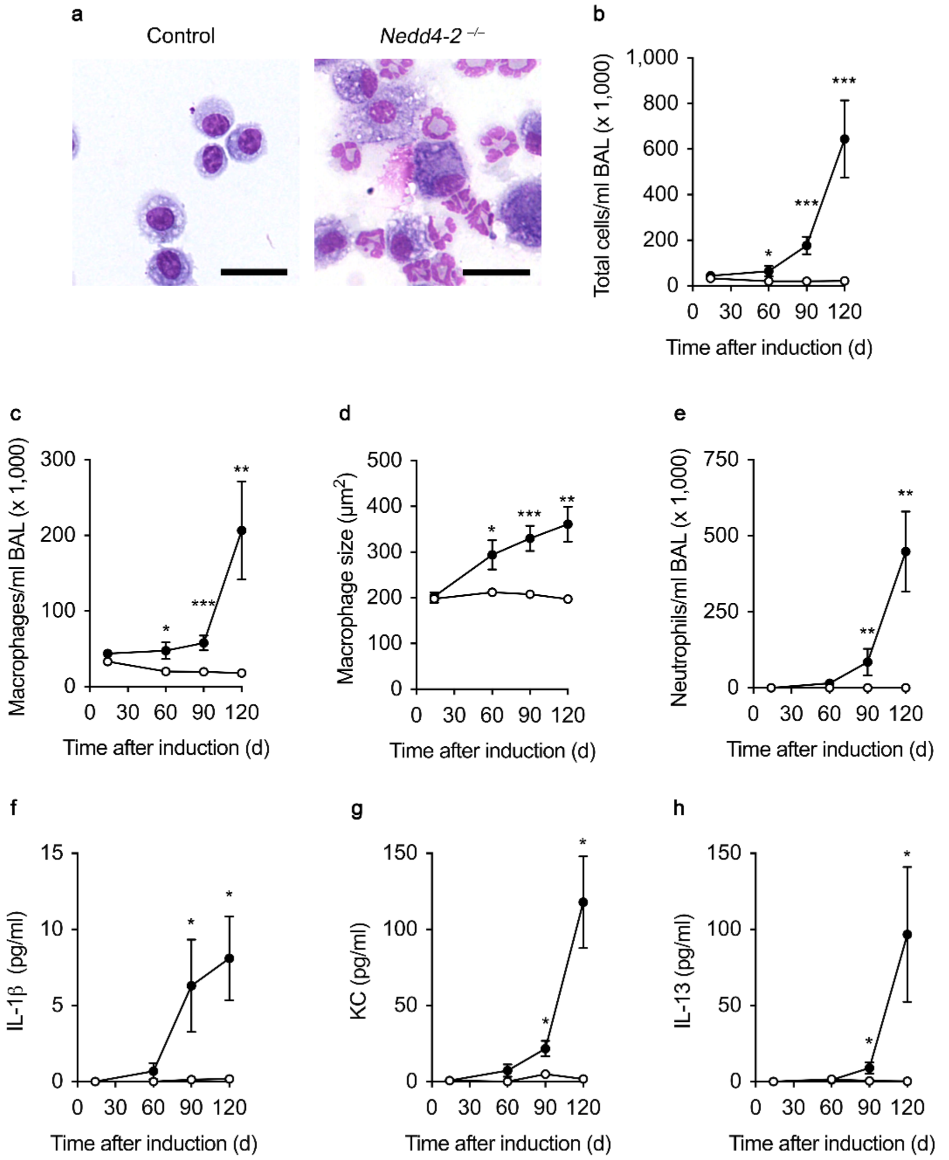

Supplementary Figure 2. Pulmonary inflammation in conditional *Nedd4-2*$^{-/-}$ mice. (**a**) Representative micrographs of BAL macrophages (scale bars, 25 µm). (**b–h**) Total cell counts (0.5, 2, 3, and 4 months, control *n* = 6, 5, 8 and 7 mice; conditional *Nedd4-2*$^{-/-}$ *n* = 5, 5, 8 and 8 mice) (**b**), number of macrophages (0.5, 2, 3, and 4 months, control *n* = 6, 5, 8, and 6 mice; conditional *Nedd4-2*$^{-/-}$ *n* = 5, 5, 8 and 7 mice) (**c**), macrophage size as marker of morphological activation (0.5, 2, 3, and 4 months, control *n* = 6, 5, 7 and 6 mice; conditional *Nedd4-2*$^{-/-}$ *n* = 5, 5, 8 and 6 mice) (**d**), number of neutrophils (0.5, 2, 3, and 4 months, control *n* = 6, 5, 8 and 6 mice; conditional *Nedd4-2*$^{-/-}$ *n* = 5, 5, 8 and 7 mice) (**e**) and concentrations of IL-1β (control *n* = 4 mice/timepoint; conditional *Nedd4-2*$^{-/-}$ *n* = 5 mice/timepoint) (**f**), KC (0.5, 2, 3, and 4 months, control *n* = 4, 4, 5 and 5 mice; conditional *Nedd4-2*$^{-/-}$ *n* = 5, 5, 4 and 4 mice) (**g**) and IL-13 (*n* = 4 mice/group) (**h**) in BAL from conditional *Nedd4-2*$^{-/-}$ mice and littermate controls after 0.5, 2, 3, and 4 months of doxycycline induction. **P* < 0.05; ***P* < 0.01 compared to control. Data are shown as mean ± S.E.M.. Source data are provided in the Source Data file.

Supplementary Figure 3

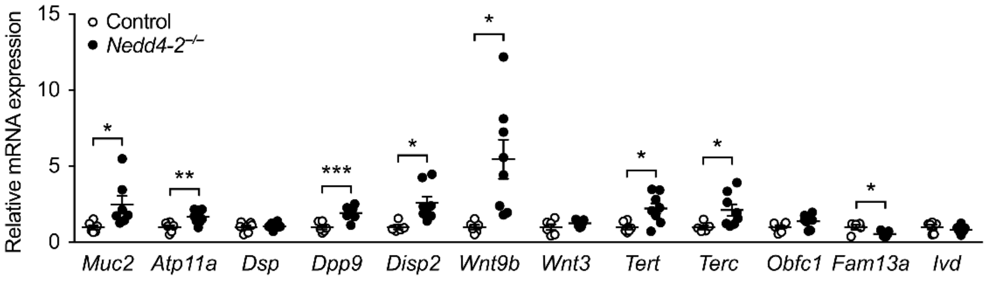

Supplementary Figure 3. Alterations in expression of genes associated with pulmonary fibrosis. Transcript analysis of whole lungs from conditional *Nedd4-2*$^{-/-}$ and control mice for 12 selected genes associated with pulmonary fibrosis in patients. Results are expressed as fold change relative to control mice. control n = 6 mice/group, except for *Disp2, Terc*, *Fam13a* n = 5; conditional *Nedd4-2*$^{-/-}$ n = 8 mice/group, except for *Muc2*, *Wnt3*, *Fam13a* n = 7 . *P < 0.05; **P < 0.01; ***P < 0.001 compared to control. Data are shown as mean ± S.E.M.. Source data are provided in the Source Data file.

Supplementary Figure 4

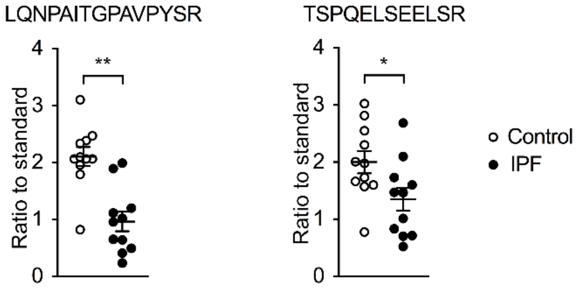

Supplementary Figure 4. Comparison of NEDD4-2 protein levels in lung biopsies from patients with IPF and controls. Peak area ratios of the two specific NEDD4-2 peptides LQNPAITGPAVPYSR and TSPQELSEELSR used for NEDD4-2 quantification were determined by parallel reaction monitoring mass spectrometry and ratio-to-standard quantification was performed for lung tissue biopsies from IPF patients and controls (n = 11/group). Ratios were obtained by dividing the cumulative peak area of the transitions of the endogenous peptide by the cumulative peak area of the transitions of the corresponding stable isotope-labeled standard peptide. *$P < 0.05$; **$P < 0.001$.

Supplementary Figure 5

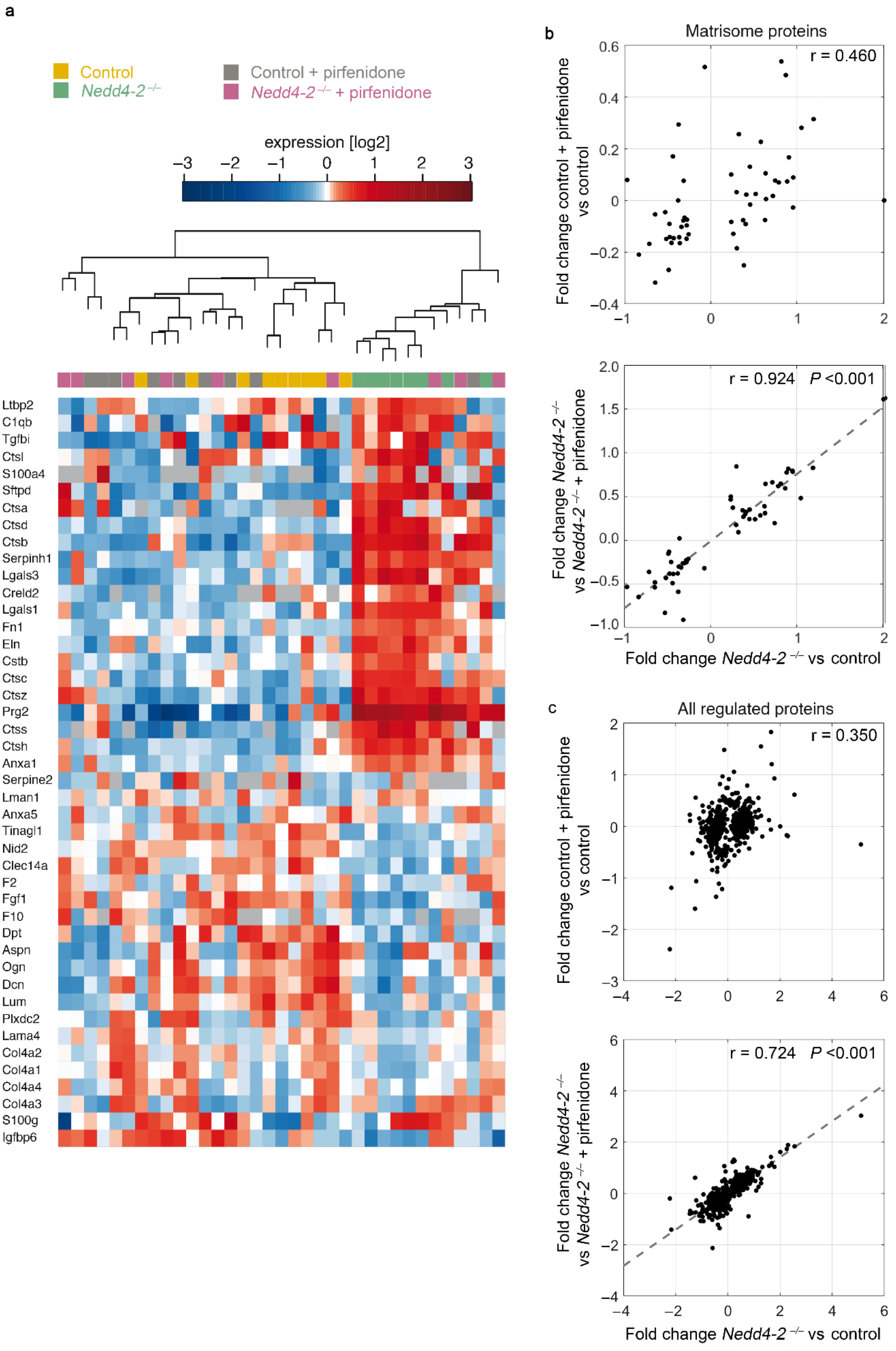

Supplementary Figure 5. Effects of pirfenidone on the lung proteome of conditional *Nedd4-2*$^{-/-}$ mice and controls. (**a**) Sample clustering based on matrisome proteins that were significantly regulated in lungs from conditional *Nedd4-2*$^{-/-}$ compared to control mice in the experiments summarized in Fig. 7a. (**b-e**) Correlation of protein fold change between control + pirfenidone versus control mice with fold change between conditional *Nedd4-2*$^{-/-}$ versus control mice (**b, d**) and correlation of protein fold change between conditional *Nedd4-2*$^{-/-}$ + pirfenidone versus conditional *Nedd4-2*$^{-/-}$ mice with fold change between conditional *Nedd4-2*$^{-/-}$ versus control mice (**c**, **e**) for regulated matrisome (**b, c**) and all regulated proteins (**d, e**). *n* = 9 mice/group and *Nedd4-2*$^{-/-}$, *n* = 8. r, Pearson correlation coefficient.

Supplementary Figure 6

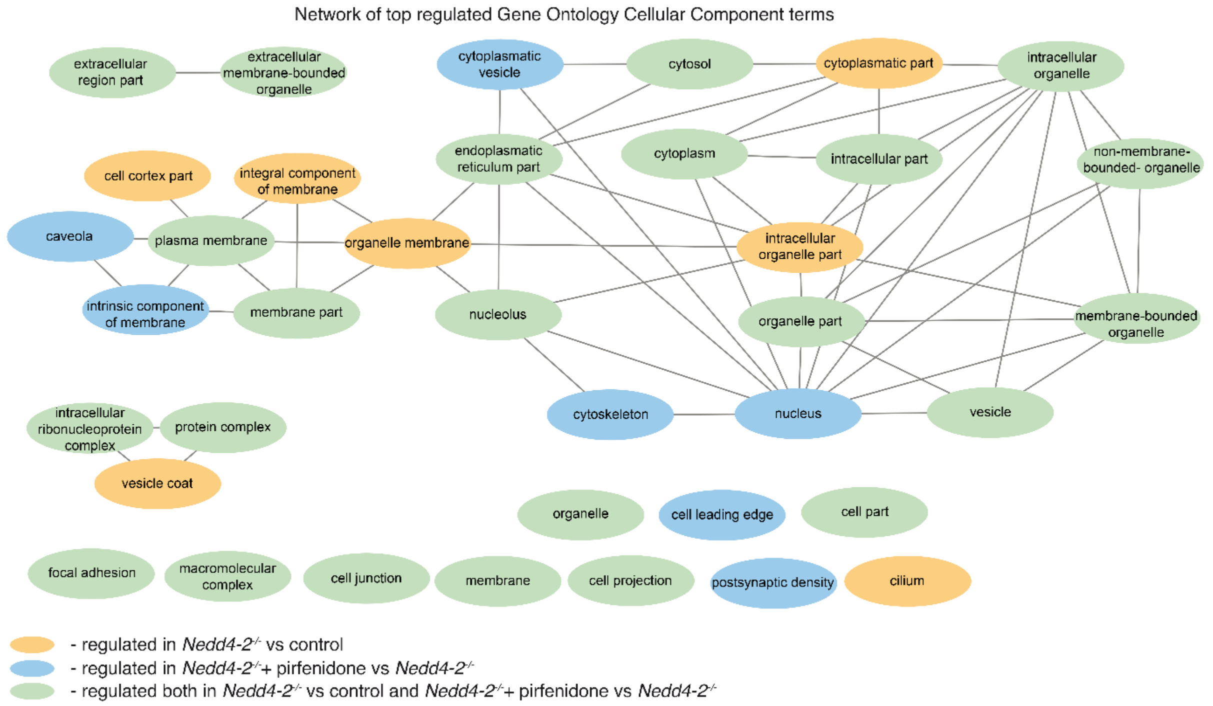

Supplementary Figure 6. Network of top regulated Gene Ontology Cellular Component Network terms. Differentially regulated proteins comparing conditional *Nedd4-2*$^{-/-}$ versus control mice and conditional *Nedd4-2*$^{-/-}$ versus conditional *Nedd4-2*$^{-/-}$ mice + pirfenidone were integrated and assigned to the Gene Ontology Cellular Component Network to illustrate different and common locations relative to cellular structures in which the differentially regulated proteins perform a function. n = 9 mice/group and *Nedd4-2*$^{-/-}$, n = 8.

Supplementary Figure 7

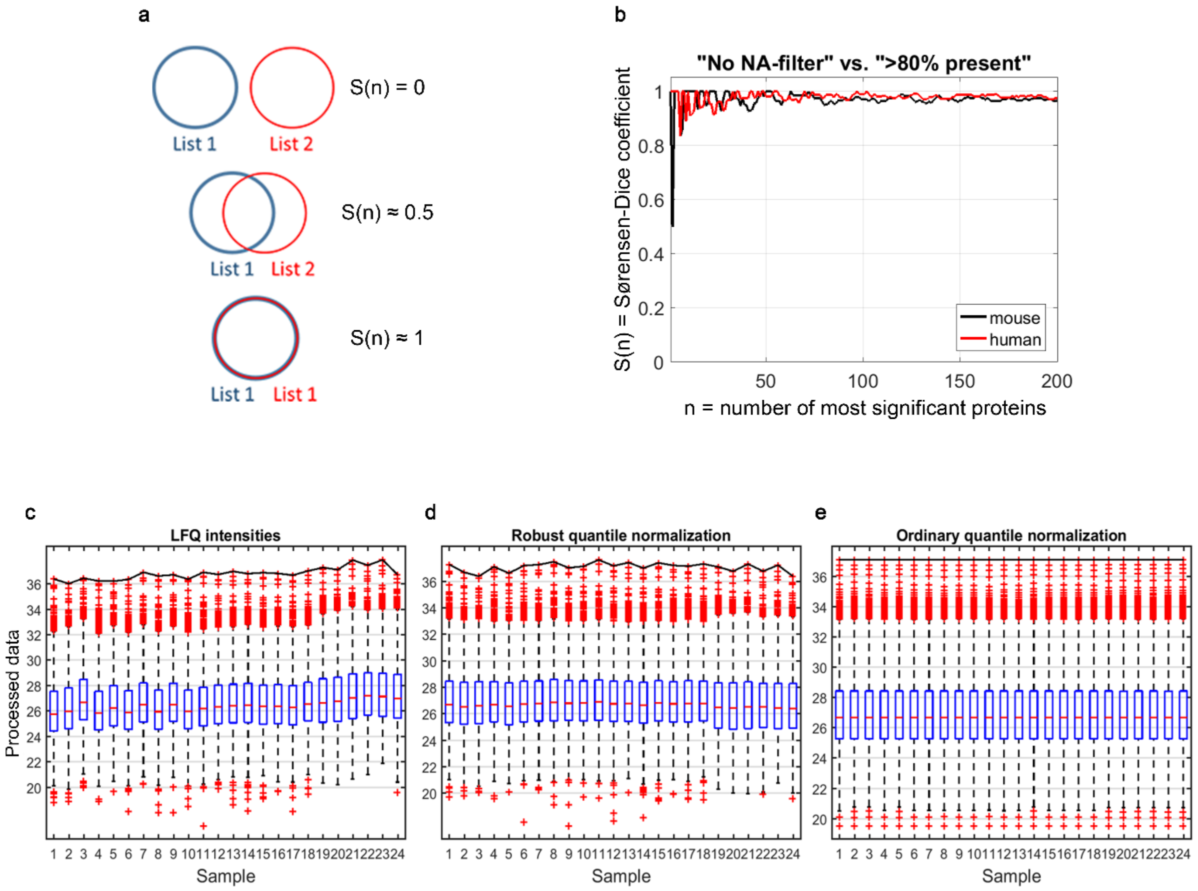

Supplementary Figure 7. Assessment of protein filtering with respect to abundance of missing values and effects of normalization. The default analysis was compared to results obtained after filtering out proteins with more than 20% missing values to ensure that this decision has only a minor impact on the results. (**a**) The 2 lists of *P*-values were sorted and the overlap of the 2 lists for the n most significant proteins was evaluated. For this purpose, the so-called Sørensen-Dice coefficient S(n) was utilized, which is defined as the relative overlap, i.e. the number of common proteins relative to the number n of considered proteins as illustrated by the examples. (**b**) The Sørensen–Dice coefficients for the two non-assigned number (NaN) filtering methods close to 1 (97% on average for the plotted range) were obtained indicating almost perfectly overlapping lists. This analysis shows that the resulting lists only weakly depend on filtering with respect to missing values for analysis of human (red line) and mouse (black line) measurements. (**c**–**e**) Illustration of the additional normalization step. Label-free quantification (LFQ) intensities (**c**) exhibited different data distributions between the samples. Therefore, an additional normalization step was applied (**d**) reducing systematic shifts of the data distributions across samples while preserving the variability in the tails. In contrast, an ordinary quantile normalization method enforces identical distributions over all samples. However, ordinary quantile normalization results in underestimation of the variability because the variance of the proteins in the distribution tails of the samples is not preserved. Serum albumin is highlighted by the black horizontal line. Since it is the most abundant protein in each sample, quantile normalization yields a vanishing variance as shown in panel (**e**). Panel (**d**) shows that our method preserves this variability.

Supplementary Tables

Supplementary Table 1. Demographics of study population

	Controls	IPF	*P* values
No. of subjects	11	11	
Age, yr			
Mean ± SD	61.2 ± 9.8	63.4 ± 7.2	
Range	42 – 73	49 – 74	
Males/females, n	7/4	8/3	
Smokers, n, (%)	8 (72.7)	8 (72.7)	
Pulmonary function test			
VC, L ± SD	3.86 ± 0.74	3.21 ± 0.81	0.065
(% predicted)	(103.9 ± 12.7)	(79.9 ± 9.8)	< 0.001
FEV1, L ± SD	2.80 ± 0.87	2.73 ± 0.68	0.840
(% predicted)	(95.7 ± 24.1)	(89.2 ± 11.4)	0.432
DLCO, mmol/min/kPa/L ± SD	1.33 ± 0.31	1.22 ± 0.18	0.414

Abbreviations: IPF = idiopathic pulmonary fibrosis; VC = vital capacity; FEV1 = forced expiratory volume in one second; DLCO = diffusing capacity of the lung for carbon monoxide

Supplementary Table 2. List of TaqMan gene expression assays used for transcript analyses

**Target gene**	**Accession No.**	**Taqman ID**
ACTB (human)	NM_001101.2	Hs99999903_m1
Actb (mouse)	NM_007393.1	Mm00607939_s1
Atp11a	NM_001293667.1	Mm00443760_m1
Disp2	NM_170593.3	Mm00467979_m1
Dpp9	NM_172624.3	Mm00841122_m1
Dsp	NM_023842.2	Mm01351876_m1
Fam13a	NM_153574.2	Mm00467910_m1
Gob5	NM_017474.2	Mm01320697_m1
Ivd	NM_019826.3	Mm00498171_m1
Muc2	NM_023566.3	Mm01276696_m1
Muc5ac	NM_010844.1	Mm01276718_m1
Muc5b	NM_028801.2	Mm00466391_m1
NEDD4-2 (human)	NM_001144964.1	Hs00971347_m1
Obfc1	NM_175360.2	Mm00614841_m1
Terc	AK085092.1	Mm01261365_s1
Tert	NM_009354.1	Mm00436931_m1
Wnt3	NM_009521.2	Mm00437336_m1
Wnt9b	NM_011719.4	Mm00457102_m1

Supplementary Table 3. List of primers used for transcript analyses by LightCycler

Target gene	Forward primer	Reverse primer
Fn1	5‘-CGGAGAGAGTGCCCCTACTA-3‘	5‘-CGATATTGGTGAATCGCAGA-3‘
G6pd	5‘-TTAAATGGGCCAGCGAAG-3‘	5‘-TGCTCTGCCATGATGTTTTC-3‘
Gapdh	5‘-AGCTTGTCATCAACGGGAAG-3‘	5‘-TTTGATGTTAGTGGGGTCTCG-3‘
Gusb	5‘-GATGTGGTCTGTGGCCAAT-3‘	5‘-TGTGGGTGATCAGCGTCTT-3‘
Hprt	5‘-CCTGGTTCATCATCGCTAATC-3‘	5‘-TCCTCCTCAGACCGCTTTT-3‘
Serpine1	5‘-AGGATCGAGGTAAACGAGAGC-3‘	5‘-GCGGGCTGAGATGACAAA-3‘
Sftpc	5‘-GGTCCTGATGGAGAGTCCAC-3‘	5‘-GATGAGAAGGCGTTTGAGGT-3‘
Sftpd	5‘-GGCCTTAAAAGGAAAACTACAGC-3‘	5‘-CCATCAGGGAACAATGCAG-3‘
Skil	5‘-GACAGGGAGGCCGAGTATG-3‘	5‘-CCGCTCCTGTCTGAGTTCAT-3‘
Smad7	5‘-ACCCCCATCACCTTAGTCG-3‘	5‘-GAAAATCCATTGGGTATCTGGA-3‘
Snai1	5‘-CTTGTGTCTGCACGACCTGT-3‘	5‘-CAGGAGAATGGCTTCTCACC-3‘
Vim	5‘-TGCGCCAGCAGTATGAAA-3‘	5‘-GCCTCAGAGAGGTCAGCAAA-3‘

Supplementary Table 4. List of scheduled peptides and precursor masses

Sequence	Mass [m/z]	CS [z]	NCE
TSPQELSEELSR (light)	688.3386	2	27
TSPQELSEELSR (heavy)	691.8472	2	27
SLSSPTVTLSAPLEGAK (light)	829.454	2	27
SLSSPTVTLSAPLEGAK (heavy)	832.9626	2	27
LQNPAITGPAVPYSR (light)	792.4306	2	27
LQNPAITGPAVPYSR (heavy)	795.9392	2	27
