# Supplementary material for: Conditional deletion of Nedd4-2 in lung epithelial cells causes progressive pulmonary fibrosis in adult mice
Source: Nat Commun. 2020 Apr 24;11:2012. doi: 10.1038/s41467-020-15743-6 (PMC7181726; doi:10.1038/s41467-020-15743-6)
Supplement: Supplementary file 3 — Description of Additional Supplementary Files [file 41467_2020_15743_MOESM3_ESM.docx]

**Description of Supplementary Files**

**File Name:** **Supplementary Data 1**

**Description:** List of differentially regulated proteins and label free quantification (LFQ) intensities in lungs of conditional *Nedd4-2^–/–^* versus control mice.

**File Name:** **Supplementary Data 2**

**Description:** List of differentially regulated proteins and LFQ intensities in lung tissue biopsies of IPF patients versus controls.

**File Name:** **Supplementary Data 3**

**Description:** List of differentially regulated proteins and LFQ intensities in lungs of untreated conditional *Nedd4-2^–/–^* mice versus pirfenidone-treated conditional *Nedd4-2^–/–^* mice and untreated controls.
