# Supplementary material for: Conditional deletion of Nedd4-2 in lung epithelial cells causes progressive pulmonary fibrosis in adult mice
Source: Nat Commun. 2020 Apr 24;11:2012. doi: 10.1038/s41467-020-15743-6 (PMC7181726; doi:10.1038/s41467-020-15743-6)
Supplement: Supplementary file 7 — Reporting Summary [file 41467_2020_15743_MOESM7_ESM.pdf]

nature research

Corresponding author(s): Marcus A. Mall
Julia Duerr

Last updated by author(s): Feb 10, 2020

Reporting Summary

Nature Research wishes to improve the reproducibility of the work that we publish. This form provides structure for consistency and transparency in reporting. For further information on Nature Research policies, see Authors & Referees and the Editorial Policy Checklist .

Statistics

For all statistical analyses, confirm that the following items are present in the figure legend, table legend, main text, or Methods section.

n/a	Confirmed	
☐	☒	The exact sample size (n) for each experimental group/condition, given as a discrete number and unit of measurement
☐	☒	A statement on whether measurements were taken from distinct samples or whether the same sample was measured repeatedly
☐	☒	The statistical test(s) used AND whether they are one- or two-sided<br>*Only common tests should be described solely by name; describe more complex techniques in the Methods section.*
☒	☐	A description of all covariates tested
☐	☒	A description of any assumptions or corrections, such as tests of normality and adjustment for multiple comparisons
☐	☒	A full description of the statistical parameters including central tendency (e.g. means) or other basic estimates (e.g. regression coefficient) AND variation (e.g. standard deviation) or associated estimates of uncertainty (e.g. confidence intervals)
☐	☒	For null hypothesis testing, the test statistic (e.g. F, t, r) with confidence intervals, effect sizes, degrees of freedom and P value noted<br>*Give P values as exact values whenever suitable.*
☒	☐	For Bayesian analysis, information on the choice of priors and Markov chain Monte Carlo settings
☐	☒	For hierarchical and complex designs, identification of the appropriate level for tests and full reporting of outcomes
☐	☒	Estimates of effect sizes (e.g. Cohen's d, Pearson's r), indicating how they were calculated

Our web collection on statistics for biologists contains articles on many of the points above.

Software and code

Policy information about availability of computer code

Data collection	Data collection is described in the Methods section
Data analysis	Data analysis is described in the Methods section

For manuscripts utilizing custom algorithms or software that are central to the research but not yet described in published literature, software must be made available to editors/reviewers. We strongly encourage code deposition in a community repository (e.g. GitHub). See the Nature Research guidelines for submitting code & software for further information.

Data

Policy information about availability of data

All manuscripts must include a data availability statement. This statement should provide the following information, where applicable:

- Accession codes, unique identifiers, or web links for publicly available datasets
- A list of figures that have associated raw data
- A description of any restrictions on data availability

All data supporting the findings of this study are available within the paper and its supplementary information files. The mass spectrometry proteomics data have been deposited to the ProteomeXchange Consortium via the PRIDE partner repository with the dataset identifiers PXD011129, PXD011120, PXD011119. For Figures 1-6 and 8 and Supplementary Figures 1-4 raw data are provided in the Source Data file.

Field-specific reporting

Please select the one below that is the best fit for your research. If you are not sure, read the appropriate sections before making your selection.

Life sciences study design

All studies must disclose on these points even when the disclosure is negative.

Sample size	Sample size was determined based on previously published data using SigmaStat version 1.01.
Data exclusions	As stated in the Methods section, outliers were identified with Grubbs' test prior to statistical calculations
Replication	Number of replicates is stated in the respective figure legend
Randomization	As stated in the Methods section mice were randomized to 2 treatment arms that received food with or without pirfenidone
Blinding	Investigators were blinded to group allocation/genotype during data collection and analysis

Reporting for specific materials, systems and methods

We require information from authors about some types of materials, experimental systems and methods used in many studies. Here, indicate whether each material, system or method listed is relevant to your study. If you are not sure if a list item applies to your research, read the appropriate section before selecting a response.

Materials & experimental systems

n/a	Involved in the study	
☐	☒	Antibodies
☒	☐	Eukaryotic cell lines
☒	☐	Palaeontology
☐	☒	Animals and other organisms
☐	☒	Human research participants
☒	☐	Clinical data

Methods

n/a	Involved in the study	
☒	☐	ChIP-seq
☒	☐	Flow cytometry
☒	☐	MRI-based neuroimaging

Antibodies

Antibodies used

rabbit polyclonal anti-MUC5B antibody H300 (Santa Cruz Biotechnology, SC-20119) at a dilution of 1:2000
mouse monoclonal anti-MUC5AC antibody 45M1 (Novus Biologicals, NBP2-15196) at a dilution of 1:1000
rabbit polyclonal anti-CCSP antibody (Upstate, 07-623) at a dilution of 1:2000
mouse monoclonal anti-acetylated-alpha-tubulin antibody 6-11B-1 (Life Technologies, 32-2700) at a dilution of 1:1000
rabbit polyclonal anti-TNC antibody (Abcam, ab108930) at a dilution of 1:1000
rabbit polyclonal anti-COL14A1 antibody (ThermoFisher Scientific, PA5-49916) at a dilution of 1:50
rabbit polyclonal anti-SERPINH1 antibody (ThermoFisher Scientific, PA5-27832) at a dilution of 1:200
rabbit polyclonal anti-Nedd4-2 antibody (Abcam, ab46521) at a dilution of 1:500
rabbit polyclonal anti-NproSP-C antibody (Beers MF et al., Am J Physiol, 1992; Beers MF et al., J Biol Chem, 1994)
mouse monoclonal anti-Smad2/3 (BD Biosciences, 610842) at a dilution of 1:2000
rabbit monoclonal anti-pSmad2/3 (Cell Signaling Technology, 9520) at a dilution of 1:2000
mouse monoclonal anti-Actb (Sigma-Aldrich, A5441) at a dilution of 1:10,000
rabbit polyclonal anti-Cnx (Enzo Life Sciences, ADI-SPA-860 at a dilution of 1:5000
biotinylated goat anti-rabbit IgG (Vectorlabs BA-1000)
M.O.M. biotinylated anti mouse IgG reagent (Vectorlabs, MKB-2225)
Alexa Fluor 488 conjugated goat anti-rabbit IgG (Jackson Immuno Research, 111-545-062)
rabbit polyclonal anti-NproSP-C raised against the Met [10]–Glu [23] domain of rat proSP-C peptide (Beers MF et al., Am J Physiol, 1995; Beers MF et al., J Biol Chem, 1994)
rabbit polyclonal anti-SP-B (PT3) raised against bovine SP-B (Beers MF et al., Am J Physiol, 1992)
rabbit polyclonal anti-SP-D (antisera 1754) raised against 2 synthetic SP-D peptides (Cao Y et al., J Allergy Clin Immunol, 2004)

Validation

All Antibodies were published and validated in previous studies. Additionally anti-Muc5ac antibody (45M1, cat. no. NBP2-15196; Novus Biologicals) was tested and validated on Muc5ac knockout tissue and anti-Muc5b antibody (H300, cat. No. SC-20119; Santa Cruz Biotechnology) was tested and validated on Muc5b knockout tissue.

Animals and other organisms

Policy information about studies involving animals; ARRIVE guidelines recommended for reporting animal research

Laboratory animals

The following mutant mouse lines were used:
B6.129/Ola;B6.Cg;B6.Cg-Nedd4L{tmBros}-Tg(Scgb1a1-rtTA)38Uhg-Tg(Luc-tetO-Cre)Bjd

	B6.129/Sv-Sftpc{tm1Swg}<br>Ages of mice are stated in the Methods section and the respective figure legends<br>Animals of both sexes were used.
Wild animals	n/a
Field-collected samples	n/a
Ethics oversight	As stated in the Methods section of the manuscript, all animal studies were approved by the animal welfare authority responsible for the University of Heidelberg (Regierungspräsidium Karlsruhe, Karlsruhe, Germany)

Note that full information on the approval of the study protocol must also be provided in the manuscript.

Human research participants

Policy information about studies involving human research participants

Population characteristics	Demographics of study population are given in Supplementary Table 1
Recruitment	Patient tissue samples were selected by staff members of the Biomaterial Bank Heidelberg (BMBH) according to confirmed diagnosis of IPF. Healthy tissues from biopsies of patients with pulmonary hamartoma served as controls and were matched to the IPF cohort for age, gender and smoking history.
Ethics oversight	patient tissue samples were obtained in accordance with the regulations of the BMBH and the approval of the ethics committee of the University of Heidelberg (approval No. 270/2001V2-V3)

Note that full information on the approval of the study protocol must also be provided in the manuscript.
